# Synthesis of crocetin derivatives and their potent inhibition in multiple tumor cells proliferation and inflammatory property of macrophage

**DOI:** 10.1186/s12906-020-2831-y

**Published:** 2020-02-03

**Authors:** Mao-Ze Wang, Jin Gao, Yang Chu, Jie Niu, Ming Chen, Qiang Shang, Li-Hua Peng, Zhi-Hong Jiang

**Affiliations:** 1 Institute of Pharmaceutics, College of Pharmaceutical Sciences, Zhejiang University, 866# Yuhangtang Road, Hangzhou, 310058 People’s Republic of China; 2 State Key Laboratory of Quality Research in Chinese Medicine, Macau University of Science and Technology, Avenida Wai Long Taipa, Macau, People’s Republic of China; 3 Livzon Pharmaceutical Group Inc., Zhuhai, People’s Republic of China; 4 National Engineering Research Center for Modernization of Traditional Chinese Medicine, Zhuhai, People’s Republic of China

**Keywords:** Solubility, Crocetin derivatives, Anti-tumor effect, Inflammation inhibition

## Abstract

**Background:**

Crocetin is a major active component of saffron, which has a wide range of pharmacological effects. However, due to its low solubility, the pharmacological effects of crocetin cannot be better utilized.

**Methods:**

In this study, we modified the chemical structure of crocetin by conjugating with ethylamine and 4-Fluorbenzylamine to enhance its solubility and biological activities. The solubility and the influence of synthesized derivatives on the proliferation of tumor cells and the inflammatory effect of macrophage were investigated.

**Results:**

It was shown that, compared with the crocetin, the synthesized derivatives have much higher solubility. Moreover, the inhibitory effect of the derivatives on varieties of tumor cells, including human ovarian carcinoma cell line, human lung cancer cell line, rat melanoma cell line was enhanced after the modification. Besides that, the derivatives were improved for the anti-inflammatory efficacy with the cytotoxicity decreased.

**Conclusions:**

The synthesized derivatives were shown for their good solubility and the great potential in tumor and inflammation treatment.

## Background

Saffron, a spice and a food colorant derived from the flower of *Crocus sativus*, is widely cultivated in Iran, Greece, Italy, Spain, India, China, Japan and other countries as an herb [1]. After collection and dehydration, it can be used mainly as the medicinal parts [2]. The history of the Saffron application in clinic can be traced back to about 2300 Before Christ [3]. Saffron contains a variety of ingredients, including carotenoids, glycosides, flavonoids, amino acids, trace elements, etc. Among them, crocetin is one of the main effective components [2], with polyunsaturated conjugate oleic acid structure. Many studies have shown that crocetin has widely pharmacological effects, which contains anti-oxidation [4], anti-cancer [5], hepatoprotection [6, 7] However, consisting of a C-20 carbon chain with seven double bonds and a carboxylic acid group at each terminal parts of the molecule, crocetin is insoluble in water. Solving this problem is the key to promote the medicinal application of this natural product. Currently, it has been reported that the use of biomaterials such as poly (lactic-co-glycolic acid) and dendrimers can improve the bioavailability of the crocetin. However, the stability and production rates are still to be improved for these biomaterials and nanotechnology based strategies [8, 9]. In our previous study [10], it has been demonstrated that the conjugation of organic compounds can help derivatives to form stable hydrogen bond by lowing solvation energy [10, 11], which in turn increases the solubility of the compounds. In present study, by conjugating the crocetin with ethylamine and 4-Fluorobenzylamine, the solubility and bioactivity of crocetin were expected to be improved. We evaluated the solubility, anti-proliferation activity and anti-inflammatory property of the synthesized derivatives. Results of this strategy is expected to provide novel evidence for the property improvement and medicinal application of crocetin.

## Methods

### Materials

1-[3-(dimethylamino)-propyl]-3-ethylcarbodiimide hydrochloride (EDCI); petroleum ether (PE); 4-fluorobenzylamine, ethylamine; 1-hydroxybenzotrizole (HOBt); Dulbecco’s modified Eagle’s medium (DMEM); Et3N, trimethylamine; the fetal bovine serum (FBS) and trypsin were purchased from the Gibco BRL (Gaithersberg, MD). BCA protein assay kit and Cell Counting Kit-8 were provided by Sangon Biotech (Shanghai) Co., Ltd. Nitric Oxide assay kit were purchased from Beyotime Institute of Biotechnology, (Jiangsu, China). The mouse macrophage cell line and the cancer cell lines were obtained from the Shanghai Institute for Biological Sciences, CAS (Shanghai). Other chemicals and reagents were of analytical grade.

### Synthesis of crocetin derivatives

**Fig. 1:**
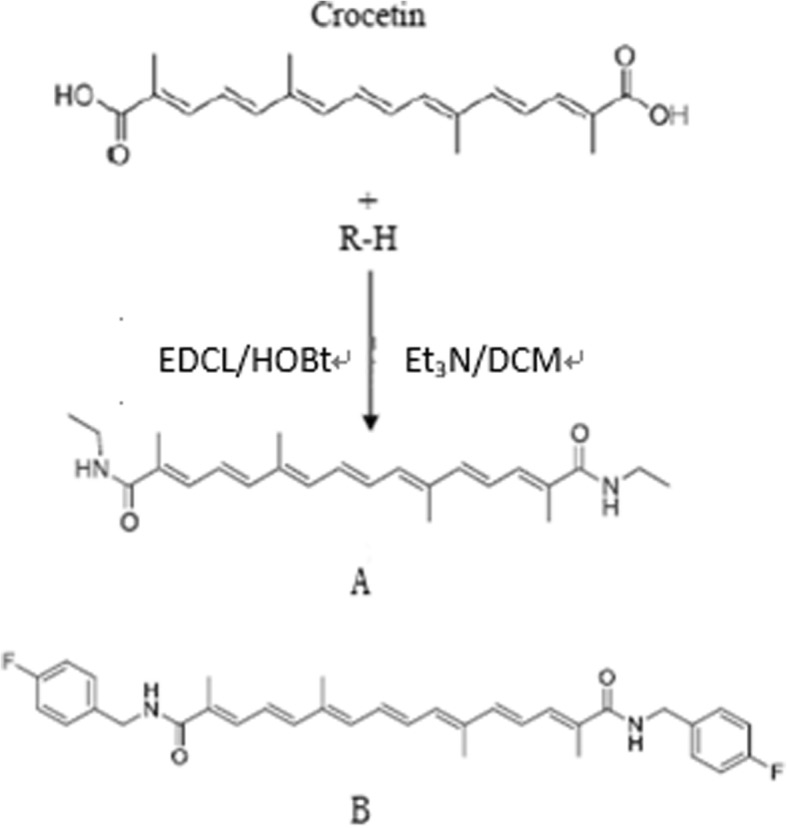
Synthesis and chemical structures of compounds A and B

The synthesis route of crocetin derivatives was showed in Fig. 1. Briefly, HOBt (2 equiv) were added to EDCI (1.5 equiv) at 0 °C, and Et_3_N (1.5 equiv) and 4-fluorobenzylamine (1 equiv) were added subsequently. The mixture solution was proceed at 0 °C for about 6 h and then at 25 °C for 12 h. After that, the mixture solution was diluted with dichloromethane. And then the unreacted organic compound in mixture was washed with 2.5% HCl and 7.5% NaHCO_3_ (15 mL, three times). The product of synthesized crocetin derivatives were obtained by evaporated under reduced pressure. Then, crocetin derivatives were purified by column chromatography.

### Solubility assays

To determine the solubility of crocetin and the synthesized derivatives in methanol, we established the standard curve by measuring its maximum absorbance with ultraviolet spectrophotometer. First, dissolving the samples in water. And then the solution was ultrasounded until the supersaturated solution was generated. After that, supersaturated solution was centrifugated at 12,000 rpm for 15 min. The supernatant of the solution was collected and freeze-dried, followed by using methanol to re-dissolve for UV detection. The concentration of the solution could be figured out by the standard curve. Upon that, we could obtain the solubility of the crocetin and its synthesized derivatives.

### Cell culture

The mouse macrophage cell line and the cancer cell lines were cultured in the DMEM medium containing 10% Fetal Bovine Serum (FBS) at 37 °C, in humidified atmosphere containing 5% CO_2_. The cell medium was replaced every two days. Once cells reached 70–80% confluence, they could be used in the next experiments.

### Anti-proliferation assay

The anti-proliferation effects of compounds on tumor cells were calculated by CCK-8 assay using Cell Counting Kit-8 (Sangon Biotech, Shanghai) according to manufacturer’s protocol. Cancer cell lines (SKOV3, A549 and B16F10 cells) were seeded in 96-well plates. 5 × 10^3^ cancer cells were added to each well and incubated overnight for proliferation. And then in test groups, they were treated with different concentrations of synthesized derivatives, which are dissolved in DMSO and diluted by cell medium. The group, whose cells were incubated without compounds, was regard as control group. After incubated for 24 h or 48 h, the cell medium was changed by fresh medium (90 μl) and 10 μl of CCK-8 assay reagent. The cells were incubated at 37 °C for 1 h. The OD values of each well were measured at 450 nm.

### Evaluation of the anti-inflammatory effect in LPS-stimulated macrophages

The anti-inflammatory effect was evaluated by using Nitric Oxide Assay kit. The RAW 264.7 cells with cell concentration 4 × 10^5^ cells/ml were seeded in a 48-well plate in DMEM containing 10% Fetal Bovine Serum and incubated overnight at 37 °C, 5% CO_2_ humidifier incubator. The cells were then treated and induced by 5 μL LPS (1 mg/mL) for 0.5 h. We used the Griess reagent to measure the NO accumulation in the supernatant. Compared with the absorbance of blank group, the NO concentration was calculated. The number of cells was calculated according to the total protein concentration analyzed by BCA assays. Thus, the anti-inflammatory effect could be calculated by the level of nitrite divide by the total protein of the corresponding well.

### Statistical analysis

Statistical analysis was performed with Origin 8.0 software (Origin Lab Inc. Massachusetts, USA). All data were expressed as mean ± standard error (SD). Comparison between two groups was done with independent sample t-test and correlation analysis. The significance was indicated by the *P* value. *P* values less than 0.05 (*P* < 0.05) were considered statistically significant.

## Result

### Solubility of the crocetin derivatives

The solubility of the synthesized derivatives was shown in Fig. 1. The results showed that the solubility in water of crocetin was 1.238 μg/mL, while the solubility in water of compound A and B were 19.55, 12.28 times more than that of crocetin, respectively (Table 1). The significant increase in the solubility of these synthesized derivatives indicated that the chemical modification of these groups, which enhanced hydrophilicity and increased hydrogen bond formation [12], is an effective strategy to increase solubility of crocetin.

**Table 1:** Water solubility of crocetin, synthesized derivatives A and B

Compounds	Wavelength (nm)	Water Solubility (μg/ml)
Crocetin	440	1.238 ± 0.001
A	421	24.20 ± 0.003
B	422	15.20 ± 0.002

### Anti-tumor effects of the crocetin derivatives

**Fig. 2:**
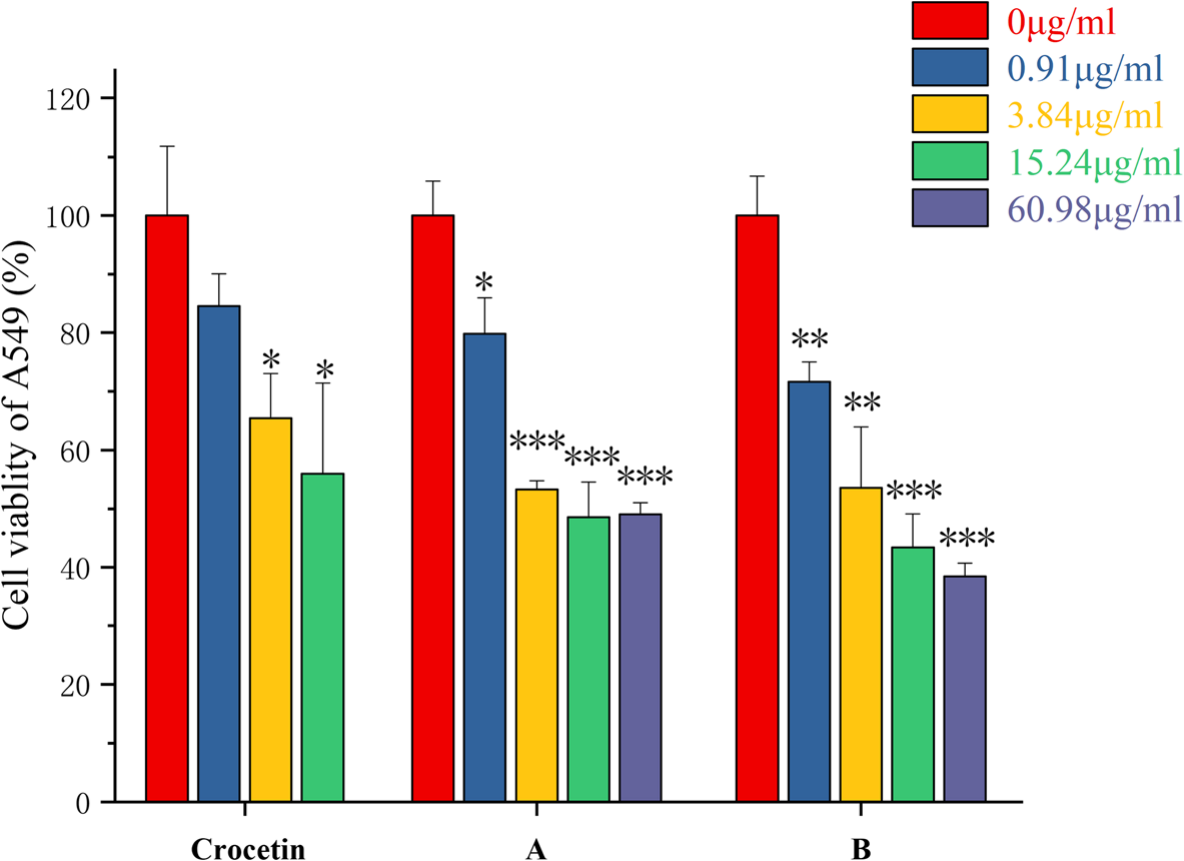
Crocetin and its synthesized derivatives A、B inhibits cell proliferation in A549 cells. A549 cells were treated with different concentrations of crocetin and its synthesized derivatives A、B inhibits. After 24 h’ co-incubation at 37 °C, the cell survival rate was determined by MTT assays. Data represent mean ± SD. The results shown were reproduced at least 6 independent experiments. *** Indicates *p* < 0.001, ** indicates *p* < 0.01, and * indicates *p* < 0.05, for each treatment group versus solvent control group

The influence of these compounds in the viability of three kinds of cancer cell lines was investigated. With different solubility, the highest concentration of the tested compounds in the mixture of water with 1% DMSO was tested as 15.24 μg/mL, 60.98 μg/ml and 60.98 μg/mL. As shown in Fig. 2, we first investigated the effects of different concentrations of crocetin and synthesized derivatives on the proliferation of A549, a lung cancer cell [13]. After one day of incubation of the compound with the cells, it was found that the cells viability was significantly decreased in contrast to that of solvent treated group. The crocetin expressed the highest suppression percentage at the 15.24 μg/mL, and the cells viability was decreased to 56%. While the maximum inhibitory efficiency of the compound A and B was expressed as 48.52 and 38.41%, at the 15.24 and 60.98 μg/mL, respectively.

**Fig. 3:**
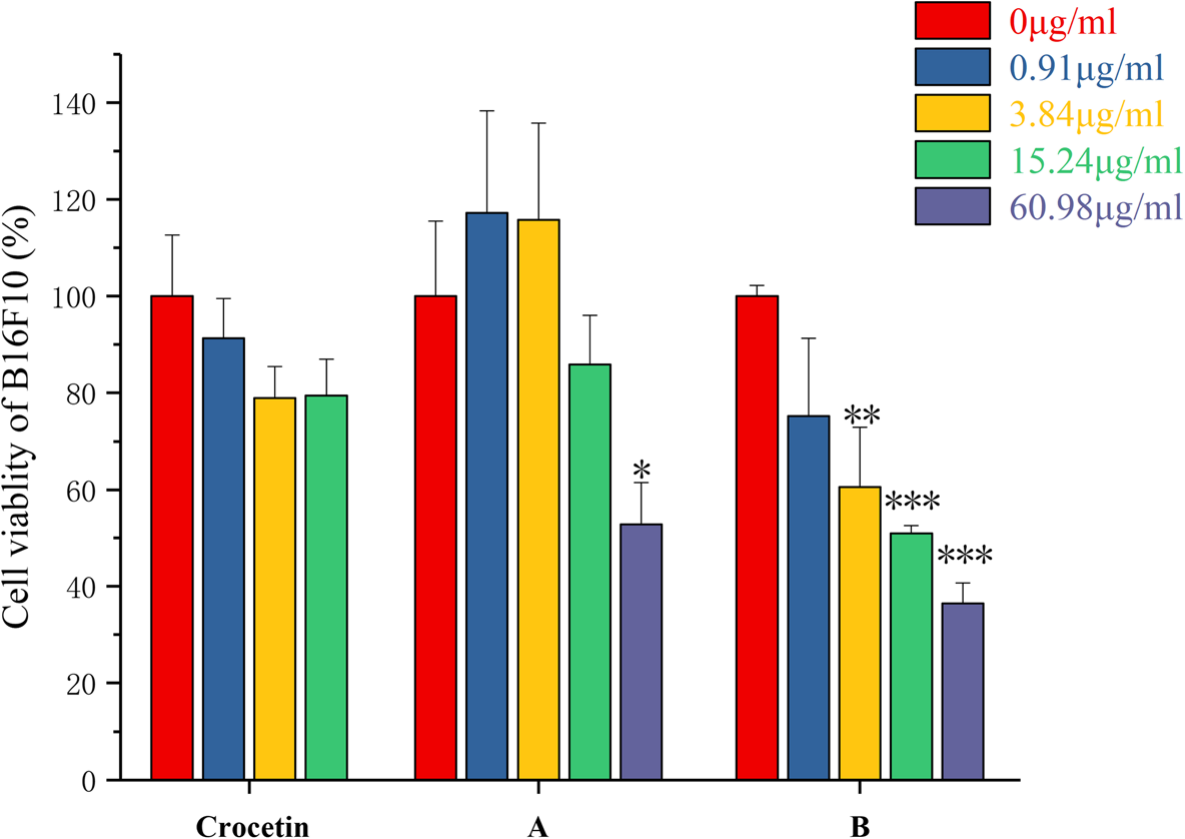
Crocetin and its synthesized derivatives A、B inhibits cell proliferation in B16F10 cells. B16F10 cells were treated with different concentrations of crocetin and its synthesized derivatives A、B inhibits. After 24 h’ co-incubation at 37 °C, the cell survival rate was determined by MTT assays. Data represent mean ± SD. The results shown were reproduced at least 6 independent experiments. *** Indicates *p* < 0.001, ** indicates *p* < 0.01, and * indicates *p* < 0.05, for each treatment group versus solvent control group

Then we investigated the influence of the compound on B16F10 cell proliferation. As shown in Fig. 3, for group treated by crocetin, the highest suppression was shown as the 21.13% at the 15.24 μg/ml. Although compound A promoted cell proliferation at concentrations of 0.91 and 3.84 μg/mL, it inhibited the proliferation with 47.14% at concentrations of 60.98 μg/mL. In the case of compound B, it showed a strong inhibitory effect on B16F10. At the concentration of 60.98 μg/mL, the inhibition rate was 65.54%.

**Fig. 4:**
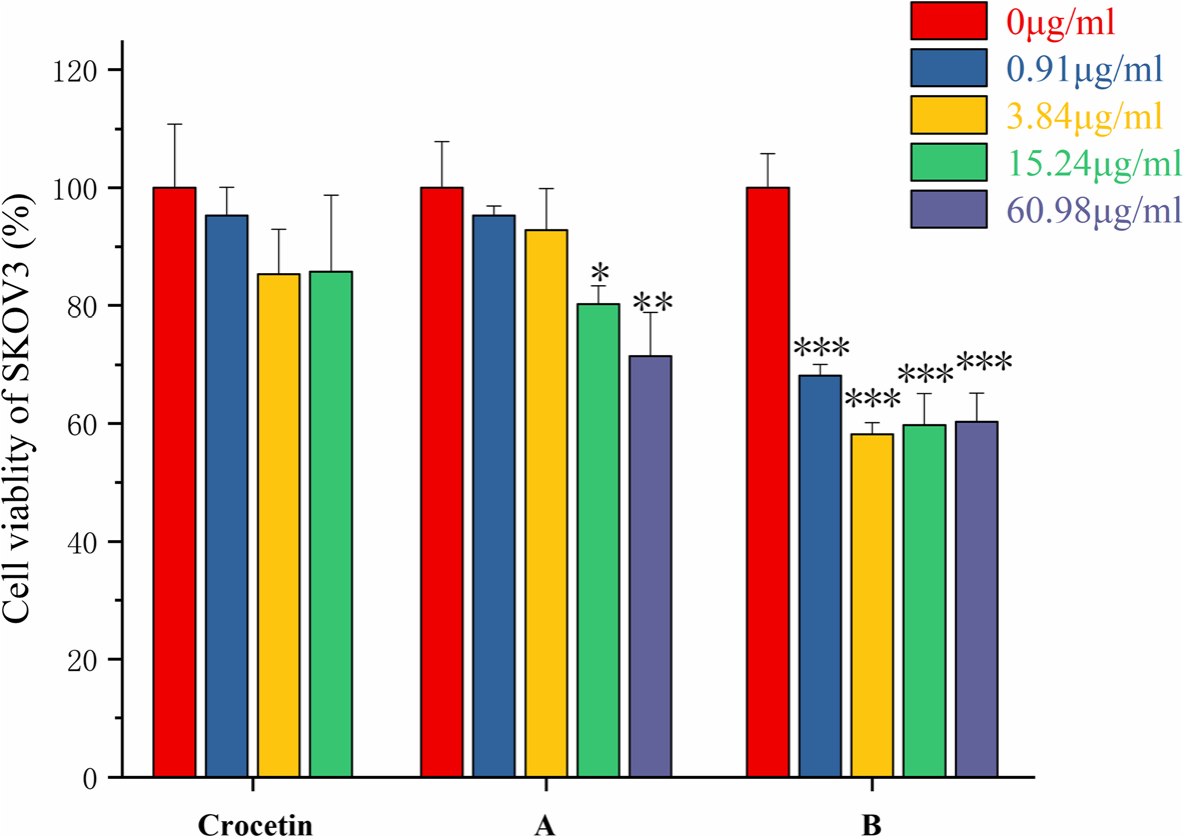
Crocetin and its synthesized derivatives A、B inhibits cell proliferation in SKOV3 cells. SKOV3 cells were treated with different concentrations of crocetin and its synthesized derivatives A、B inhibits. After 24 h’ co-incubation at 37 °C, the cell survival rate was determined by MTT assays. Data represent mean ± SD. The results shown were reproduced at least 6 independent experiments. *** Indicates *p* < 0.001, ** indicates *p* < 0.01, and * indicates *p* < 0.05, for each treatment group versus solvent control group

As shown in Fig. 4, we also investigated the effect of the compound on SKOV3 cells. Compared with the crocetin, the compound A, B showed the enhanced cancer inhibition efficacy of 28.53, 41.85% at 60.98, 15.24 μg/mL.

These results indicated that crocetin and its derivatives have a good inhibitory effect on the proliferation of the tested tumor cells. With the increased solubility of the derivatives, the anti-tumor activity of the crocetin was obviously improved. The inhibitory effect of compound on A549 and B16F10 cells was significantly higher than that of crocetin. For SKOV3 cells, there was no dose-dependent effect. But the overall inhibition efficacy was strong. As shown in Table 2, compound B had the lowest IC50 value, which indicated that it had the strongest anti-proliferation effect in the tested tumor cells. Results of this study proved the scientific evidence for that the modification of N atoms with hydrophilic groups is a promising strategy, which enhances the solubility and the biological effects of crocetin and has potential application for the anti-tumor and anti-inflammation.

**Table 2:** IC50 values of the synthesized compounds in different tumor cell lines

IC50	A549 (μg/ml)	B16F10 (μg/ml)	SKOV3 (μg/ml)
crocetin	55.39	270.13	559.0
A	49.59	63.98	168.31
B	6.23	15.08	–

### Anti-inflammatory effect of the crocetin derivatives

**Fig. 5:**
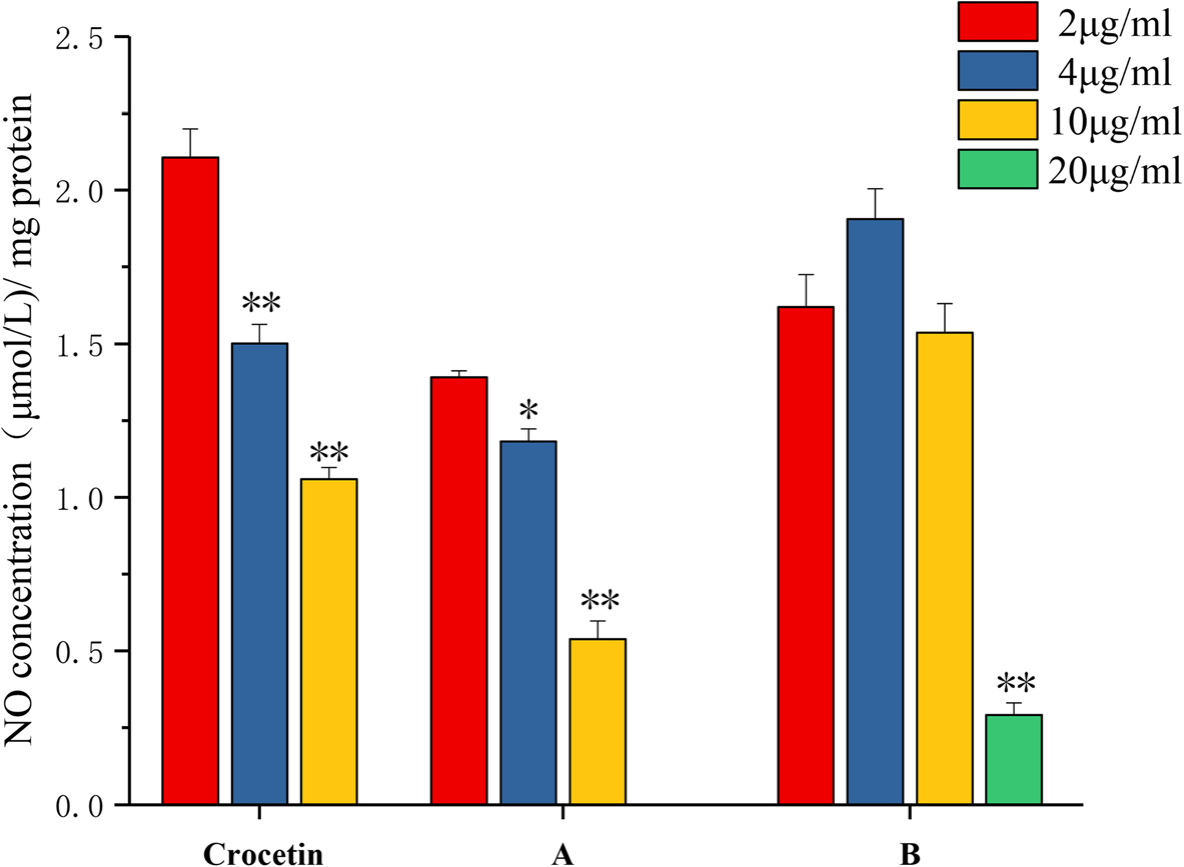
Influenced of crocetin and its synthesized derivatives A、B on NO production in RAW 264.7 cell. RAW 264.7 cells were treated with different concentrations of crocetin and its synthesized derivatives A、B inhibits. After 24 h’ co-incubation at 37 °C, the expression of NO was measured by Griess reagent and protein levels were calculated by the BCA assays. Data represent mean ± SD. The results shown were reproduced by at least 6 independent experiments. ⁠** Indicates *p* < 0.01 for each treatment group compared with 2 μg/ml group; * indicates *p* < 0.05 for each treatment group compared with 2 μg/ml group

Oxidative stress is one of the critical steps that induce a lot of disease, including inflammation. Despite that various studies have reported the anti-oxidative effect of saffron, there are few study on the anti-inflammatory effects of crocetin to be reported [14]. In our study, the anti-inflammatory potential of crocetin and its derivatives were investigated. Large amounts of nitric oxide (NO) are positively correlated with the degree of inflammatory response [15]. Therefore, we determined the effect of compound at 2、4、10、20 and 40 μg/mL on the NO concentration in LPS activated macrophage cells to indicate their anti-inflammatory potential. As shown in Fig. 5, the level of NO in the macrophage was significantly reduced by the crocetin or derivatives treatments. When the compound A treatment at the 20 μg/mL, the NO concentration was under the detection limit. NO could not be detected in the compound B at the 40 μg/mL treated cells. The anti-inflammatory effect of compound A was dose-dependent. These results indicated that crocetin compounds have great significance in research and application.

### Cytotoxicity of the crocetin derivatives

**Fig. 6:**
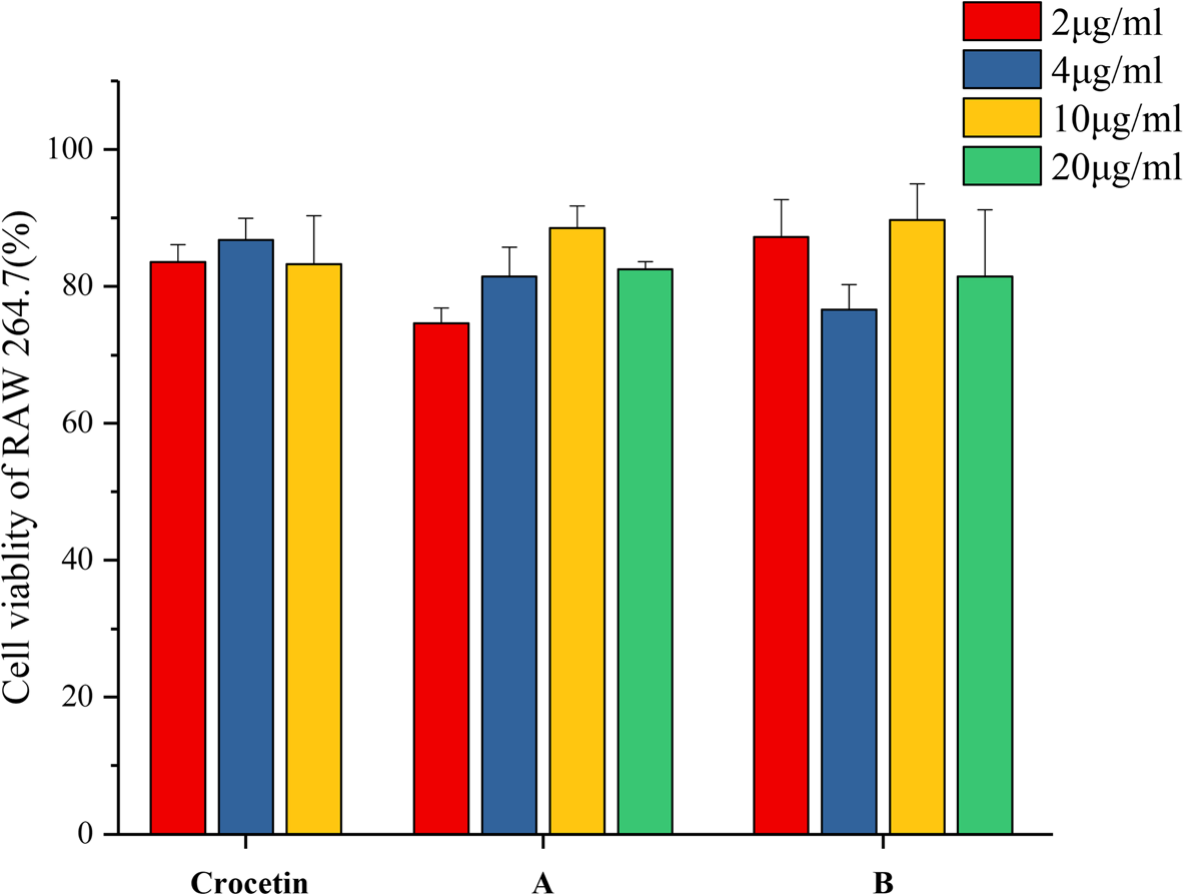
Cytotoxicity of crocetin and its synthesized derivatives A、B in RAW 264.7 cells. Total protein concentration of the cells were determined by BCA assays. Data represent mean ± SD. The results shown were reproduced by at least 6 independent experiments. ⁠** Indicates *p* < 0.01 for each treatment group compared with 2 μg/ml group; * indicates *p* < 0.05 for each treatment group compared with 2 μg/ml group

In order to investigate whether the inhibition in the level of NO can be induced by cytotoxic effect, we examined the viability of RAW 264.7 cells treated with the compound by CCK-8 assays. As shown in Fig. 6, the tested compounds expressed no significant toxicity to the macrophage cells at all the tested concentration.

## Discussion

The major pharmacological component of saffron, crocetin possesses many pharmacological activities. However, due to its low water solubility, the clinical applications of crocetin is scarce [16]. It was reported that a large number of solvents have to be added to dissolve the compound and/or loaded onto other drug delivery vehicles. But these methods have limitations, such as side effects or complicated procedures. At the same time, few studies have reported the strategies that modifying the chemical structure of crocetin to improve its solubility. In this study, we investigated the influence of the conjugation of crocetin with the ethylamine or 4-Fluorbenzylamine in the solubility and biological activities of the derivatives. The results showed that this strategy have significantly not only increased the solubility, but also improved the anti-tumor properties and anti-inflammatory potential of the derivatives. Particularly, on the basis of retaining the structure with polyenoid as the core, the synthesized derivatives exert its pharmacological activities without obvious cytotoxicity to normal cells induced, indicating that the synthesized derivatives have a selective anti-proliferation effect on tumor cells that has great potential for in vivo application.

In mammals, oxidative stress and inflammatory responses cause major defense networks that help cells adapt to changes in the environment [4, 17–19]. However, chronic oxidative stress or inflammation is one of the most significant risk factor for the development of various human diseases including cancer, diabetes, neurological diseases [20–22]. The relationship between inflammation and cancer has been a hot area to be investigated for decades. The results of our study showed that the crocetin derivatives can effectively decrease the level of nitric oxide (NO) secretion, one of the most important inflammatory cytokines. At the same time, the test result of cytotoxicity of the synthesized compounds in the mouse macrophage cell line showed that the crocetin derivatives do not cause any damage to the cells, indicating the specific inhibition of the crocetin derivatives in NO secretion. Bathaie et al. reported that crocetin might interact with DNA and cause certain conformational changes, making the DNA unstable. In addition, they found that the crocetin significantly inhibits the expression of matrix metalloprotein (MMPs) in tumor cells, which is related to tumor invasion and its metastasis [23–25]. The anti-proliferation effects of the crocetin derivatives identified in this study might be contributed to the inhibition of the tested compounds in the process of synthesizing DNA dependent RNA polymerase II enzyme, biomacromolecule [23–25]. The anti-inflammatory potential of the compounds might also contribute to the anti-tumor potential of the derivatives. The significantly increased solubility of the derivatives might also be a major contribution to the improved efficacy.

## Conclusion

This study demonstrated that the conjugation of ethylamine or 4-Fluorbenzylamine with the crocetin is the novel and hopeful strategy to enhance the solubility, anti-tumor properties and anti-inflammatory potential of crocetin. The synthesized derivatives are shown as the hopeful therapeutic agents with good solubility and vigorous bioactivities in the tumor and inflammation treatment.

## Data Availability

All data generated or analysed during this study are included in this article.

## Abbreviations

BCA: Bicinchoninic acid
CCK-8: Cell counting kit-8
CDCl3: Deuterochloroform
CHCl3: Chloroform
DCM: Dichloromethane
DMEM: Dulbecco’s modified eagle’s medium
DMSO: Dimethyl sulphoxide
EDCI: 1-(3-dimethylaminopropyl)-3-ethyl carbodiimide
Et3N: Trimethylamine
EtOAc: Ethyl acetate
FBS: Fetal bovine serum
HCl: Hydrochloric acid
HCl: Hydrochloride
HOBt: 1-Hydroxy, benzotriazole, hydrate
iNOS: Inducible nitric oxide synthase
LPS: Lipopolysaccharides
MeOH: Methanol
NaHCO3: Sodium bicarbonate
NO: Nitric oxide
PE: Petroleum ether
SD: Standard deviation
